# Editorial: Anesthetic-induced neurotoxicity and neurocognitive impairment of vulnerable brains

**DOI:** 10.3389/fnagi.2023.1293491

**Published:** 2023-10-10

**Authors:** Shuang Zeng, Jiaqiang Zhang

**Affiliations:** Department of Anesthesiology and Perioperative Medicine, People's Hospital of Zhengzhou University, Henan Provincial People's Hospital, Zhengzhou, Henan, China

**Keywords:** sevoflurane, apoptosis, neurodegeneration, calcium, tau

With the development of technology, more and more infants are undergoing general anesthesia for surgery, other interventions, or clinical examinations in the early stages of life. However, whether general anesthesia affects the function and structure of the developing infant brain remains an important, complex, and controversial issue. Sevoflurane is the most commonly used anesthetic for infants, but this drug has potential neurotoxicity. Short term or single exposure to sevoflurane has a weaker impact on cognitive function, while long-term or repeated exposure to general anesthetics may lead to cognitive dysfunction. However, the underlying mechanisms are still being debated, and preventive measures need to be further explored. To provide an overview of this topic, we have selected six original research and review articles that delineate sevoflurane and developmental neurotoxicity.

Apoptosis is the main pathway leading to the death of nerve cells, and most preclinical studies of sevoflurane have regarded apoptosis as the main cause of neurotoxicity. This type of programmed cell death is normal during development, but long-term exposure to sevoflurane in young animals can lead to a 50-fold increase in nerve cell apoptosis rates (McCann and de Graaf, 2017). Hu et al. (2019) found that sevoflurane induces apoptosis of nerve cells in the developing brain of young rats through a cascade of apoptosis regulated by brain-derived neurotrophic factor (BDNF), which also led to a decrease in the anti-apoptotic/pro-apoptotic ratio, and induced intrinsic apoptosis.

Neurodegeneration is the foundation of many cognitive disorders. In some studies, exposure to sevoflurane can cause neurodegenerative changes. In a study by Wu et al. (2020) sevoflurane significantly up-regulates SIRT-2 in newborn rat hippocampus, promotes the expression of pro-inflammatory/M1-related markers in microglia, and activates microglia. Microglial activation has also been implicated in neurodegenerative diseases (Yeh et al., 2017). Iron is essential for normal neuronal function. However, excess iron has been linked to several neurodegenerative diseases. Sevoflurane exposure disrupts iron homeostasis and leads to hippocampal iron overload, which leads to neurodegenerative disease (Wu et al., 2020). Repeated exposure of neonatal mice to 3% sevoflurane induces tau phosphorylation Yu et al. (2020), leading to tau accumulation and the formation of neurofibrillary tangles, a signature pathology of the neurodegenerative brain, suggesting tau plays a key role in the neurodegenerative brain (Pirscoveanu et al., 2017; Yang and Wang, 2018). The neurodegenerative changes caused by neonatal sevoflurane exposure may involve multiple pathways, but the exact mechanisms are unknown and require further study.

Calcium imbalance can lead to nerve cell damage. Calcium plays a crucial role in human physiology, especially in the central nervous system (CNS). The precise maintenance of Ca^2+^levels is crucial for normal cell function, and dysregulation of calcium homeostasis can lead to neuronal cell damage. Sevoflurane exposure leads to a significant decrease in calcium concentration in the ER, followed by a significant increase in Ca^2+^ in the cytoplasm and mitochondria through overactivation of the IP3 receptor (Yang et al., 2008). Mitochondrial Ca^2+^overload leads to mitochondrial respiratory injury, ROS activation, ATP reduction, and MOMP, thereby inducing neuronal damage and apoptosis (Danese et al., 2017; Yang and Wei, 2017). Neonatal exposure to 2% sevoflurane upregulates CA1 region Ca^2+^ activated potassium channel type 2 (SK2s), which has persistent detrimental effects on long-term depression (LTD) and long-term enhancement (LTP) (Yu et al., 2018). Calcium homeostasis is an important mechanism of neurotoxicity induced by sevoflurane anesthesia, which is related to mitochondrial dysfunction, astrocyte dysfunction and neuronal dysfunction. Maintaining intracellular calcium homeostasis may be an effective intervention for sevoflurane induced developmental neurotoxicity.

Sevoflurane exposure in the developing brain reduces intermediates in glucose metabolic pathways, including lactic acid, significantly lower levels of succinic acid, and significantly reduced total creatine pools, including high-energy creatine phosphate and creatine (Liu et al., 2015). Alterations in amino acid metabolism in the neonatal brain may also play a key role in sevoflurane induced neurotoxicity. Exposure to sevoflurane during rapid brain development in monkeys also promotes microglial activation, which can be detected by upregulated transporter (TSPO) expression (Zhang et al., 2016). Activated microglia are a major source of cytokines in the central nervous system and release a range of pro-inflammatory cytokines and chemokines, such as monocyte chemokine-1, interleukin, macrophage colony-stimulating factor, tumor necrosis factor (TNF)-α, and macrophage inflammatory protein-1 α/β. Maternal exposure to sevoflurane directly affects fetal glial cells and enhances IL-6 through phosphorylated erk signaling (Hirotsu et al., 2019). Neuroinflammation is involved in sevoflurane-induced developmental neurotoxicity, which may be an important target for further study of sevoflurane-induced developmental neurotoxicity.

Tau protein is a microtubule-associated protein, and excessive tau phosphorylation promotes the formation of insoluble tau aggregates. Tau phosphorylation leads to cognitive dysfunction in mice (Faraco et al., 2019). Tao et al. (2014) found that Mice 6 days after birth were exposed to 3% sevoflurane for 2 h per day to induce tau phosphorylation through GSK-3β activation multiple times (rather than single), thereby increasing levels of IL-6 in the hippocampus and decreasing levels of PSD-95, leading to cognitive impairment. While these effects of sevoflurane did not occur in the tau KO mice, suggesting that tau plays a role in sevoflurane induced neuroinflammation and synaptic defects in mice.

In summary, the aim of the present Research Topic was to explore the potential mechanisms of sevoflurane and developmental neurotoxicity and the potential measures to prevent its occurrence in preclinical and clinical settings. These manuscripts provide important new insights on the topic. In the future, we hope that more studies will be performed to examine the association between sevoflurane and developmental neurotoxicity.

## Author contributions

SZ: Writing—original draft. JZ: Writing—review and editing.
